# Lockdown in France: Impact on Families of Young Children With Special Needs

**DOI:** 10.3389/fpsyg.2022.781030

**Published:** 2022-04-26

**Authors:** Stéphanie Pinel-Jacquemin, Amalia Martinez, Maud Martinasso, Valerie Katkoff, Thiébaut-Noël Willig, Chantal Zaouche Gaudron

**Affiliations:** ^1^Laboratory LISST-CERS, Université Toulouse Jean Jaurès, Toulouse, France; ^2^Scientific Interest Group “Bébé, petite Enfance en COntextes,” GIS BECO-Université Fédérale de Toulouse Midi-Pyrénées, Toulouse, France; ^3^Occitadys, Toulouse, France; ^4^Paediatrics Unit, Clinique Ambroise Paré, ELSAN and Eventail 31, Toulouse, France; ^5^Association Française de Pédiatrie Ambulatoire, Orléans, France

**Keywords:** lockdown, families, young children, special needs, parental competence, COVID-19, developmental difficulties

## Abstract

**Background:**

Families with young children have faced serious challenges during the first lockdown as a result of the COVID-19 pandemic. In addition to remote working, parents have had to monitor their children’s schoolwork and manage their daily lives. When one of the children also has neuro-developmental disorders, this results in an increased burden. We can therefore wonder how these families with one or more young children (under 6 years old) with special needs have experienced and dealt with this lockdown.

**Aim of the Study:**

In this context, the “COVJEUNENFANT” study focused more specifically on the subjective experience, as a parent, of those who cared for children with special needs (i.e., with developmental disorders, neurodevelopmental disorders, proven disabilities or chronic health conditions) compared to the general population. We wished to see if the consequences of the health crisis were significantly different from those perceived by respondents in the general population (*n* = 490) and if the sociodemographic structure of these families differed from those of other respondents.

**Methods:**

Ninety three French families with at least one child under 6 years old and one with developmental difficulties or a chronic illness, from a cohort of 490 control families, participated in a web-based survey during the first lockdown, from the 28th April 2020 to 29th May 2020.

**Results:**

After presenting the participants’ sociodemographic characteristics, the results show that these French families (*n* = 93) are less wealthy than the control population “without special needs” (*n* = 397), have felt more pressures originating from their environment (families, friends, colleagues, media, social networks…), have suffered from more health issues (other than COVID-19), have taken more measures to protect themselves (social-distancing), and were less likely to feel happy. A significantly larger number of them lamented the lack of free time and voiced a larger need for information regarding children’s education. However, their parental role was felt as being more satisfying and their family relations strengthened more than in the general population of participants.

**Conclusion:**

It is apparent that urgent prioritisation is needed in order to support and care for these families by continuing to provide care for their children in one way or another, and by ensuring that their need to adapt again does not exceed their own abilities and resources, especially as young children, who have high levels of requirements, are present in the home.

## Introduction

Children with developmental disorders benefit from special measures provided by society to acknowledge and compensate for their hardship, and to ensure their access to equal rights in terms of education, health and every other aspect of their lives. In France, public policies have been designed over decades. However, those who might benefit from these policies might present vulnerability factors which go beyond the direct consequences of the child’s disorders (for example mobility, sensory, cognitive disorders), social and economic deprivation, single parenthood, etc.

The first lockdown following the official COVID-19 world pandemic announcement lasted 55 days in France, from March 17 to May 11, 2020. French residents had to remain at home, except for a maximum of 1 h per day whilst following social distancing rules.

Many international scientific studies have covered its consequences on the psychosocial, cognitive and functional wellbeing of children and teenagers (Shah et al., 2020), students (Roux et al., 2021), adults (Rossi et al., 2020), parents and non-parents (Morelli et al., 2020) and the elderly (Morales-Vives et al., 2020). Few studies have focused on the experience of families with one or more young children (under 6 years old) with special needs (Bobo et al., 2020; Cacioppo et al., 2020). For example, Bobo et al. (2020) gathered information about the wellbeing and global life conditions of French children and adolescents with ADHD (mean age: 10.5) whereas Cacioppo et al. (2020) identified potential healthcare issues relating to the wellbeing of French children with physical disabilities (mean age: 9.5) and Nonweiler et al. (2020) assessed emotional and behavioural difficulties during the COVID-19 pandemic in English children with neurodevelopmental disorders. While these studies highlighted a higher prevalence of emotional symptoms, behavioural issues and fewer prosocial behaviours etc., none of these aforementioned studies had focused on the parental experience, or they had done so while focusing on one aspect of life during lockdown, for example the impact of homeschooling (Bobo et al., 2020).

More recently, Bentenuto et al. (2021) investigated parental stress, co-parenting, and child adjustment in Italian families with children aged between 3 and 17 years with neurodevelopmental disorders (*N* = 82) and typical developing children (*N* = 82). Their results showed a significant increase in parental stress and children externalising behaviours, but not in co-parenting, although they did not distinguish between families of very young children and others. Results from Burnett et al. (2021) also showed higher levels of distress for parents of children with a neurodevelopmental disorder compared to those of typically developing children. However, once again the children in the study were over 3 years old and from Australian and Italian populations.

While parents are usually the main providers of support, care and covering the fundamental needs of the children, young children are also cared for or treated in specialised institutions (Zaouche Gaudron et al., 2021-to be published). Yet, during lockdown, most of the socialisation and care institutions were closed. In a family where parents must already manage their jobs, their older children’s education and care for a child with neurodevelopmental difficulties, the presence of young children aged between zero and 6 years old who rely on their families and require constant monitoring can create an additional burden for those parents. In a study by Rogers et al. (2021) which aimed to identify the ways eight English mothers of children with intellectual disabilities managed, only one had a child under 6 years old. While these authors showed that mothers experienced increased burden and stress, the results did not, once again, point out the increased difficulties for those families with young children. Regarding this increased burden, the literature highlights an important phenomenon occurring during the COVID-19 outbreak: the so-called “parental related exhaustion”—defined as feelings of being overextended and depleted of one’s emotional and physical resources. Exhaustion is a key aspect of parental burnout, which occurs due to prolonged exposure to parental stress, which in turn influences the psychological wellbeing of their children. Indeed, in their study on parental-related exhaustion during the COVID-19 pandemic, involving 1226 Italian families with at least one child aged 0–13 years, Marchetti et al. (2020) found that parenting-related exhaustion was predicted by psychological distress, lower parental resilience, motherhood, fewer perceived social connections, marital status (being single), having a child with special needs, having a large number of children and having younger children.

The main objective of the “COVJEUNENFANT” programme^1^ was therefore to analyse what parents of young children with one child with neurodevelopmental disorders had experienced, felt, and what resources they have sought in order to respond to a unique situation during the lockdown. The aim was to document the positive and negative impacts of this crisis for families and their young children. We have worked toward creating a “real-time” research project (Zaouche Gaudron et al., 2020). In doing so, we wished to study specifically the ways in which families already facing societal constraints have adapted in this unstable period, compared to the general population, and how this pandemic situation has negatively impacted them. Our hypotheses are that they are facing more difficulties, and experience more pressure—due, among other things, to the interruption of care for their child—and tensions within their family compared to families in the general population. These families experience more fear for themselves and their children, along with more negative feelings and are more isolated. In short, their life during lockdown is more complex than that of parents with young children within the general population.

We will therefore focus on French families with at least one child under 6 years old and including at least one child with either developmental disorders, neurodevelopmental disorders, a confirmed disability or a chronic health issue. Developmental disorders cover motor, language, intellectual and cognitive development.

## Materials and Methods

### Participants

#### Population Without Special Needs (Control Population)

Four hundred and ninety families responded to our questionnaire (of the participants, 88% were mothers and 12% fathers; Table 2). 40% were between 35 and 39 years old. 95% were in a relationship. 39.5% had one child, 43.1% two children and 17.4% more than three children. This population was well educated, with 65% of participants and 49% of their partners having studied 4 years in higher education. Most of them (41.8%) had a median income within the second tercile (1979.19€) and 59.4% felt relatively wealthy. They had comfortable professions, with 37% executives and 29.5% in an intermediate profession. 8.3% were unemployed (5% among the partners). Finally, 17.1% have seen a decrease in their income during lockdown and 50.9% of the participants’ partners worked from home.

**TABLE 1A:** Care provided to children with and without developmental disorders (DD).

	Care without a declared DD	Care with DD	DD without care	Total families with special needs
Number of families	53	35	5	93
		40 families DD		
	88 families with care			

*DD, Developmental Disorder.*

**TABLE 1B:** Ages of the 40 children diagnosed with a developmental disorder.

Children with developmental disorders	1–2 years	2–3 years	3–4 years	4–5 years	5–6 years	More than 6 years	Total
Child 1	1	1	2	5	5	12	26
Child 2	0	2	0	0	4	4	10
Child 3	1	2	0	0	1	0	4
Total	2	5	2	5	10	16	40
	under 6 years: 60%					40%	100%

**TABLE 2:** Sociodemographic characteristics: bivariate analyses of “special needs” population vs. “population without special needs.”

		“Special needs” population *N* = 93 (18, 98%)		Population “without special needs” *N* = 397 (81, 02%)		Chi-squared test
Explanatory variables		N	%	N	%	*Fisher’s exact test *P* value < 0.05
Marital situation	Couple	83	89.2	377	95.0	0.039
	Single	10	10.8	20	5.0	
Single-parent home	No	83	89.2	380	95.7	0.014
	Yes	10	10.8	17	4.3	
Number of children living in lockdown	1 child	26	28.0	157	39.5	0.002
	2 children	36	38.7	171	43.1	
	3 children and over	31	33.3	69	17.4	
Respondent’s educational level	High school diploma or lower	12	12.9	29	7.3	0.076
	Less than 4 years after high school diploma	31	33.3	110	27.7	
	4 years or more after high school diploma	50	53.8	258	65.0	
Partner’s educational level	High school diploma or lower	25	26.9	60	15.1	0.001
	Less than 4 years after high school diploma	25	26.9	106	26.7	
	4 years or more after high school diploma	32	34.4	209	52.6	
	Not specified	11	11.8	22	5.5	
Income terciles based on consumption units (INSEE)	Median income 1st tercile (1257.56€)	43	46.2	122	30.7	0.003
	Median income 2nd tercile (1976.19€)	38	40.9	166	41.8	
	Median income 3rd tercile (2857.14€)	12	12.9	109	27.5	
Financially comfortable	Not at all	28	30.1	67	16.9	0.014
	Relatively	48	51.6	236	59.4	
	Very	17	18.3	94	23.7	
Occupational category	Farmer/craftsmen/business owners/accredited professionals	5	5.4	25	6.3	0.058
	Executive	28	30.1	147	37.0	
	Intermediate professions	20	21.5	117	29.5	
	Employees/workers/assistants	27	29.0	75	18.9	
	Unemployed	13	14.0	33	8.3	
Partner’s occupational category	Farmer/craftsmen/business owners/accredited professionals	9	9.7	33	8.3	0.092
	Executive	27	29.0	168	42.3	
	Intermediate professions	17	18.3	75	18.9	
	Employees/workers/assistants	21	22.6	78	19.6	
	Unemployed	8	8.6	20	5.0	
	Not specified	11	11.8	23	5.8	
Has your income decreased during lockdown?	No	69	74.2	329	82.9	0.054
	Yes	24	25.8	68	17.1	
Where did your partner work during lockdown?	Mainly outside the home	28	30.1	93	23.4	0.022
	Mainly at home	31	33.3	202	50.9	
	Didn’t work	23	24.7	73	18.4	
	Not specified	11	11.8	29	7.3	

*Only significantly different results are listed in this table.

#### Special Needs Population

Among these 490 families who answered the questionnaire, 93 declared having a child with a disability or developmental difficulties (19% of respondents, among which 8.6% fathers and 91.4% mothers) (Table 2). 89.2% lived as a couple within the same home. A quarter of the adult respondents were under 35 years old, 40.9% between 35 and 39 years old and a third were over 39 years old (*m* = 36.7; σ = 4.7). Compared to the rest of our control population, these families have more children (a third of them have three children or more), have lower financial means (51.6% state that they are “relatively” comfortable and 30.1% “not at all” comfortable financially, compared to respectively, 59.4 and 16.9% of the population without special needs, *p* = 0.014). Regarding employment, 30.1% of respondents are executives, 29.0% are employees or workers, and 21.5% from intermediate occupations. 14% declared being unemployed. Furthermore, before lockdown, 51.6% worked full-time and 36.6% worked part-time. As for their partners, 71% worked full-time, and only 9.7% worked part-time. Of those 93 families, 40 have at least one child with a developmental disorder^2^ (DD) (representing 43.0% of those respondents and 8.2% of the overall respondents), affecting the older child in one quarter of the families (Table 1A). The others mentioned that they received care for other reasons (general medicine, speech therapy, physiotherapy, psychomotricity…),^3^ either at home or outside the home. Around a quarter of them (23.7%) were able to keep on receiving care during lockdown, either fully, partially, or through different means.

Sixty percent of the children with a developmental disorder are under 6 years (Table 1B).

The parents of the remaining 53 families were not asked to specify which of their children had health issues.

### Instrument and Procedure

From April 28, 2020 to May 29, 2020, a questionnaire was sent via a dedicated website^4^ and through the study’s various partner networks (For example academic, professional, friends, families), Facebook, Twitter, LinkedIn, etc., to families with at least one child under 6 years old living in France and/or its overseas territories. This questionnaire was designed for the purposes of this study and has not yet been scientifically approved. Several questions were taken from a more generic questionnaire about social relations and solidarity during lockdown which was created by teacher-researchers from the CNRS.^5^

The questionnaire was made of 69 questions as follows (see Supplementary Material for more details on questions 27–69):

1) *General information* (questions 1–18) : Socio-demographic data, children’s specific medical or paramedical care, continuity of current care and the existence of a developmental disorder (motricity, language, school learning, intellectual) in one of the children.

1) *Parents’ experience of the pandemic* (questions 19–26)

An open question invites the participant to express themselves freely with regards to this “unusual” time in order to better understand how they and their young children experienced this lockdown (in terms of activities, play, screen time, diet, schoolwork, education in a broad sense…) and how they felt (Q19). The following questions asked them to describe “four main events” in “yesterday’s day in lockdown” (Q20), specifying for each event whether they were alone or with someone, and if so who, during that day (Q21), and specifying how they felt among a list of options (Looking forward to finishing; Happy; Frustrated/annoyed; Depressed; Feeling competent/qualified; Feeling mistreated/abused; Warmth/friendship; Anger/hostility; Worried/anxious; Feeling pleased; Feeling criticised; Fatigue) and how intensely they felt the emotion (from not at all to a lot) (Q22–25). Question 26 allowed us to learn how often (from never to often) they found it hard to control important things in their lives, if they felt confident in their ability to handle their personal problems, if they felt like things were going as they wanted and if they felt as though difficulties were accumulating to the point where they could not handle them.

2) *Child’s self-expression* (question 27)
3) *Activities and daily life during lockdown* (questions 28–46)
4) *Accommodation during lockdown* (questions 47–58)
5) *Employment since the beginning of lockdown* (questions 59–68)
6) Question 69 was an open question inviting respondents to add *any comments which they felt were important.*

*Questions specific to families with children with special needs* were:

– Question 8: Is one of your children usually receiving care, either at home or outside the home, for medical issues (general medical care, speech therapy, physiotherapy, psychomotricity, etc.) aside from routine appointments?
– Question 9: If one of your children receives specific medical and paramedical care (apart from routine care), did it continue during lockdown?
– Question 10: Among your children, if any of them have a developmental disorder (motricity, language, learning, intellectual), which child is it: child 1, child 2…

The time required to complete the questionnaire was estimated at thirty to 45 min (sometimes lasting up to 1 h). The respondents were also asked to confirm that they had read and understood the information displayed on the website’s consent page, that the research team had answered all their questions satisfactorily and that they had been made aware that they were free to withdraw their consent at any time while taking part in the study without any negative impact for anyone in their family. This study was approved by the General Data Protection Regulation’s Data Protection Officer (RGPD-CNRS [TRRECH-502]) and by the research ethics board (CER; number 2020-290) from the Université Fédérale de Toulouse Midi-Pyrénées.

### Statistical Analysis

From questions 8, 9, and 10, we created a sample representing the families (as explained earlier). The indicators from the gathered data were recoded in order to obtain variables which were relevant both in terms of the numbers and in relation to the health and socio-economic context at the time, such as: a variable describing the cultural level of households based on the qualifications of the respondents and their partners, a variable showing the net monthly income per consumption unit divided into terciles, and a variable relating to the social distancing measures recommended during this health crisis.

This sample was described and groupings of socio-demographic variables were made to provide a better understanding and relevance of the analyses.

In this article we will focus on the variables specific to the impact of lockdown on some aspects of the life of families with young children with developmental disorders (*n* = 93): fraternal relationships (questions 28–31), social distancing (question 35: gloves, masks, coveralls, one meter distance, washing hands several times per day, hydroalcoholic gel, disinfecting door handles/switches…) along with daily tasks, educational tasks, support received for the children, free time, time outdoors, changes in family relations, family pressure, parenting perceptions and the need for information (questions 32–46). We wished to see if the consequences of the health crisis are significantly different from those perceived by respondents in the general population (*n* = 490) and if those families differ from those of other respondents in their sociodemographic structure, such as one describing the cultural level of the households from the respondents’ and their partners’ qualifications, or a variable showing the net monthly income by consumption units split into terciles. We then compared the subpopulation of households with at least one child with a developmental disability with the “control population” (the population which did not have a child with a developmental disability), using the chi-square test. If the conditions for this test were not met, Fisher’s exact test was performed. We have checked the raw residuals of chi-squared tests with more than one degree of freedom (see Supplementary Appendices 1, 2). We tested the influence of the characteristics of participants and their partners, the way they experienced lockdown, their environment and their employment through this comparison. We consider 0.05 as an alpha risk and the threshold for statistical significance in bivariate analyses.

Finally, we add three logistic regression models with respondents’ behaviours during lockdown (M1), respondents’ characteristics (M2) and both behaviours and characteristics (M3) (see Table 3B).

**TABLE 3A:** Experience of families with a child with special needs: bivariate analyses of “special needs” population vs. “population without special needs.”

		“Special needs” population *N* = 93 (18, 98%)		Population “without special needs” *N* = 397 (81, 02%)		Chi-squared test
Explanatory variables		*N*	%	*N*	%	*Fisher’s exact test *P* value < 0.05
Health issues other than COVID-19	No	56	60.2	300	75.6	0.003
	Yes	37	39.8	97	24.4	
Type of pressure felt from relatives	No pressure	23	24.7	158	39.8	0.016
	1 or 2 slight pressures or 1 strong pressure	36	38.7	137	34.5	
	At least 2 strong pressures or at least 3 slight pressures	34	36.6	102	25.7	
Wore protective gloves when leaving the home	No	71	76.3	333	83.9	0.086
	Yes	22	23.7	64	16.1	
Maintained social distance when leaving the home	No	14	15.1	24	6.0	0.003
	Yes	79	84.9	373	94.0	
Wore a mask when leaving the home	No	36	38.71	164	41.31	0.646
	Yes	57	61.29	233	58.69	
Washed clothes after having left the home	No	58	62.4	298	75.1	0.013
	Yes	35	37.6	99	24.9	
Disinfected door handles in the home	No	62	66.7	311	78.3	0.017
	Yes	31	33.3	86	21.7	
Washed hands with hydroalcoholic gel to protect their children from contamination	No	65	69.9	316	79.6	0.043
	Yes	28	30.1	81	20.4	
Washed clothed after having left the home to protect their children	No	62	66.7	310	78.1	0.020
	Yes	31	33.3	87	21.9	
Number of protective actions (apart from social distancing rules) used to avoid contamination when leaving the home	None	38	40.9	223	56.2	0.008
	1–4 protective actions	55	59.1	174	43.8	
Do you lack free time?	No, not at all	12	12.9	92	23.2	0.025
	Yes, I really lack time	53	57.0	170	42.8	
	Yes, I somewhat lack time	28	30.1	135	34.0	
Were you happy during lockdown?	No	33	35.5	95	23.9	0.022
	Yes	60	64.5	302	76.1	
Did the relationships among your family change?	They got more tense	24	25.8	95	23.9	0.021
	They didn’t change	20	21.5	143	36.0	
	They got stronger	49	52.7	159	40.1	
Did the relationship between the parents change?	They got more tense	10	10.75	58	14.61	0.002
	They didn’t change	20	21.51	143	36.02	
	They got stronger	20	21.51	40	10.08	
	Other type of relationship	43	46.24	156	39.29	
Did the relationship between the children and the parents change?	They got more tense	14	15.05	37	9.32	0.031
	They didn’t change	20	21.51	143	36.02	
	They got stronger	29	31.18	119	29.97	
	Other type of relationship	30	32.26	98	24.69	
Your perception of your role as a parent during lockdown	Satisfying	65	69.9	214	53.9	0.018
	Neutral	18	19.4	108	27.2	
	Not satisfying	10	10.8	75	18.9	
Need information regarding education	No	68	73.1	334	84.1	0.013
	Yes	25	26.9	63	15.9	
Struggle to obtain staple foods	No	61	65.59	335	84.38	0.000
	Yes	32	34.41	62	15.62	
Care still provided for the child	No	70	75.27	382	96.22	0.000
	Yes	22	23.66	0	0.00	
	Not specified	1	1.08	15	3.78	

**TABLE 3B:** Multivariate models regarding the links between behaviours and characteristics of respondents and the presence of a child with a DD within the household during the first lockdown.

	Odds ratio	Std. Err.	*P* < z	[95% Conf.	Int.]	Odds ratio	Std. Err.	*P* < z	[95% Conf.	Int. J]	Odds ratio	Std. Err.	*P* < z	[95% Conf.	Int.]
	Final Ml: behavioural variables during lockdown					Final M2: Respondents’ characteristics					Final M3 : Respondents’ behaviour and characteristics				
**Types of pressures felt during lockdown (reference category: slight pressures)**															
Average pressures	1.50	0.45	0.176	0.833	2.702						1.43	0.43	0.243	0.784	2.611
Strong pressures	1.97	0.60	0.027	1.080	3.583						1.93	0.59	0.034	1.052	3.543
**Health Issues other than COVID-19 experienced during lockdown (reference category: no)**															
Yes	1.70	0–42	0.035	1.038	2.776										
**Difficulty in obtaining staple foods (reference category: no)**															
Yes	2.48	0.66	0.001	1.473	4.183						2.54	0.692	0.001	1.491	4.335
**Number of children in the home during lockdown (reference category: 1 child)**															
2 children						1.69	0.50	0.060	0.939	3.041	1.45	0.430	0.206	0.813	2.597
3 children or more						3.92	1.29	0.000	2.055	7.479	3.24	1.061	0.000	1.713	6.162
**Partner’s education level (reference category: high school diploma}**															
Less than 4 years in higher education						0.63	0.21	0.176	0.322	1.230					
4 years or more in higher education						0.39	0.12	0.004	0.205	0.747					
Missing						1-34	0.62	0.517	0.546	3.322					
**Financial means (reference category: not at all comfortable)**															
Rather comfortable financially						049	0 15	0020	0.271	0.896	0 42	0.12	0.004	0-234	0.753
Very comfortable financially						0.50	019	0.064	0.237	1093	0 42	0.15	0.022	0.203	0.884

All of these analyses were processed through the STATA software (StataCorp LP, College Station, TX, version 16.1).

## Results

The analysis of bivariate tests show significant differences at various levels: among sociodemographic characteristics of those families and among their experience of this first lockdown (Tables 1A, 3A).

### Sociodemographic Variables

Comparing our sample population with the control population highlights some socio-economic differences (Table 2). First this population shows more single-parent households than in the control population (10.8 vs. 5%; *p* = 0.014). Similarly, they had a significantly higher number of children (one third had three children vs. 17.4% within the control population; *p* = 0.002). We can also notice that fewer of the participants and their partners in the sample population have studied over 4 years after high school than in the control population (*p* = 0.076 for participants and *P* = 0.001 for their partners, with 7% of missing data). Furthermore, around a quarter of the people in our sample had a drop in their income during lockdown following temporary measures supported by the government (25.8%), 16.1% were put on leave, close to one out of five have seen their working hours reduced (19.4%; *p* = 0.321) and close to one out of four mentioned a deterioration of their working conditions (24.7%; *p* = 0.952), which doesn’t differ significantly from the control population.

Respondents were more likely to be unemployed (14 vs. 8.3% in the control population, *p* = 0.058), similarly to their partners (8.6 vs. 5%, *p* = 0.092). Finally, 45.2% of respondents worked from home during lockdown, along with 33.3% of their partners (vs. 50.9% in the control population; *p* = 0.022). Only 15.1% of the respondents and 30.1% of their partners worked outside the home. Among the 10.75% of single-parent households, 60% did not work during lockdown and 40% worked mainly from home. The data regarding housing did not differ significantly from that of the control population (type of accommodation, number of rooms…).

The median income based on consumption units falls significantly more often within the first tercile (46.2 vs. 30.7% of control families: *p* = 0.003), which explains why a significantly larger number of those families declare that they are “not at all” financially comfortable (30.1% of families with special needs vs. 16.9%; *p* = 0.014), even though the occupational categories of the respondents and their partners do not differ. A quarter of those households have seen their income reduced during lockdown, compared to 17.1% of the households in the control population [χ^2^(1) *p* = 0.054].

### Experience of Lockdown Variables

#### Difficulties and Parental Pressure

Only one difficulty appears to be significantly different from the control population: *find staple foods:* it was also mentioned by 34% of them, compared to 16% of families in the control population [χ^2^(1) *p* = 0.000] (Table 3A). While other important difficulties are mentioned, such as noise in the home (11.83% of participants) or tensions with other people living there (20.43%) and lack of space (10.75%), they are not significantly different from the control population.

Regarding their experience of lockdown, and generally speaking, families of a child with neurodevelopmental disorders have felt *more pressure* (*p* = 0.016) and have *suffered* from more health issues (other than COVID) than others (*p* = 0.003).

Families with special needs felt a considerably higher *amount of pressure* (partner, family, friends, colleagues, educators or teachers, the media, social media, health professionals or social services) than the control population (36.6 vs. 25.7% of respondents, *p* = 0.016) and felt that they really didn’t have enough *free time* (57 vs. 42.8% of respondents; *p* = 0.025).

Our logistic regression model number 1 (Table 3B) focusing on behaviours during lockdown, shows that: the respondents who have felt strong pressures, compared to those who have felt slight pressures from their relatives, are nearly twice as likely to be in a household where one of the children had a developmental disorder (DD) (OR = 1.97) compared to households without a child with DD. Furthermore, the risk of experiencing pathologies other than COVID-19 during lockdown is multiplied by 1.70 for families of a child with DD. Finally, the risk of facing difficulties in obtaining staple foods is doubled in an household with a child with DD (OR = 2.48).

The final model number 2 describes the characteristics of the respondents’ households. We can notice that the higher the number of children in a household, the more the probability of having a child with a DD increases, up to 3.92 times higher for households with 3 children. The education level of the partner, along with the financial situation of the household seem to be protecting factors for children with a DD, with a risk multiplied by 0.39 in situations where the partner has completed 4 years or more of studies in higher education and halving the risk for respondents who feel that they are in a very comfortable financial situation (OR = 0.5).

In the final model number 3, we kept only the significant variables from the two previous models. This provides us with a model combining the two previous ones. Therefore, the presence in the household of a child with a DD is a risk factor for feeling pressure during the first lockdown, with a risk multiplied by 1.93. Difficulties in obtaining staple foods are similar, as the risk of being in a household with a child with a DD is then multiplied by 2.54. A similar observation can be made regarding the number of children within the household: as that number increases, the risk also increases, reaching 3.24 for households with 3 children or more. Finally, the financial situation seems to be a protective factor, with a reduced risk for respondents claiming to be “relatively comfortable” or “very comfortable” financially (OR = 0.42).

We did not find a significant difference regarding *“negative” feelings* such as fatigue, irritability, stress, worry and anxiety, which were at least as present as within the control population (data not shown). Nevertheless, over a third of families stated that they were *not happy* during the first lockdown, compared to 24% of respondents in the control population (*p* = 0.022). However, *their role as a parent* appeared to be more satisfying than within the control population (*p* = 0.018), despite the fact that almost one out of three households with at least one child with special needs (26.9%) needed some *information regarding education* (following rules, setting limits…) vs. 15.9% of households within the control population (*p* = 0.013).

#### Relationships Within the Family

In terms of family, the *relationships within the family* got stronger between parents and children or between partners for over half of the households with at least one child with developmental disorders [52.7% vs. 40.1% for control families (*p* = 0.021)] (Table 3A). Those relationships got more tense for close to a quarter of the families [25.8 vs. 23.9%, χ^2^(2) *p* = 0.021]. However, for 21.51% of parents from a household with a child with (neuro)developmental impairments, the relationship between partners got stronger, compared to 10.8% within the control families. Furthermore, relationships between children and parents got stronger for close to a third of families, both with a child with a disorder and within control families.

#### Protective Measures

Despite this, it must be noted that a significantly lower number of them followed *social distancing rules* outside the home (84.9 vs. 94% of the total population; *p* = 0.003) (Table 3A). Wearing masks outside was not yet mandatory during this first lockdown and was not significantly different between families with and without special needs (*p* = 0.646). However, those families did take more *measures to protect themselves* (other than social distancing rules): when parents left the home during lockdown, almost 60% of them, vs. 44% for households without special needs [χ^2^(3) *p* = 0.008], took between 1 and 3 measures to protect themselves from the spread of the virus, among which: wearing gloves, washing their clothes after leaving the home, disinfecting door handles and wearing coveralls.

## Discussion

Our study looked at the way French parents in families with a child with neuro-developmental disorders and at least one young child under 6 years old experienced lockdown. The data from our online study provides information both on the socioeconomic profile of those families and on the positive and negative impact this lockdown had on them. This lockdown was made harder for these families by the lack of autonomy of young children and the need to keep up with care for the child with a neuro-developmental disorder and schoolwork.

### A Population of Less Educated and Less Financially Comfortable Parents

The study of socioeconomic variables evidences a difference in the socioeconomic level of respondent families affected by child disability or chronic illness compared to other families in the study. Faced with the worries regarding the future caused by the lockdown, being less educated and feeling less financially comfortable could also explain the increase in anxiety and in negative feelings reported by these families. Spinelli et al. (2020) found a similar link between socioeconomic variables and individual stress (measured through the *Depression Anxiety Stress* scale; *p* < 0.05) in their study of 845 Italian families with children between 2 and 14 years old. Zahaika et al. (2021, p. 5) suggest that “education can positively influence the caregivers’ self-confidence in dealing with different life situations, and how they behave in response to health problems faced by their child.” This could explain why the parents in our study have felt that they needed further information regarding their children’s education. A differential study between parents of children with developmental difficulties, some of them rather wealthy and others less so, could provide further elements of interest.

### An Improved Ability to Adapt to Stress, or More Difficulties and Suffering?

Our hypotheses were that these families experienced more difficulties and pressures, and felt more fear and negative feelings than the families of young children in the control population.

Our hypothesis regarding the increased *difficulties* encountered was, however, not entirely confirmed. Indeed, similarly to single-parent households (Moscaritolo et al., 2021), only resourcing difficulties came on top of health issues (other than COVID), suggesting an additional burden for the most vulnerable families. Other difficulties (noise, tensions, lack of space, etc.) were not significantly different from those felt by the control population.

While most studies conducted in France during the first lockdown focused on estimating the extra psychological distress felt by children with (neuro)developmental disorders (Bobo et al., 2020), the study titled “COVJEUNENFANT,” conducted during that same period, asks parents about perceived benefits and difficulties. As one could expect, our results show that the families of young children with a disability [(neuro)developmental disorder, chronic illnesses] had suffered more and perceived *more pressures* during the lockdown: our study specifies that these pressures originate particularly from the people around them and from a lack of time. Rogers et al. (2021, p. 2) also point out that “the longer the hours of care dedicated to a child with intellectual disabilities and the higher their level of dependency, the higher the levels of strain experienced by the carer.” The types of stress experienced by parents as identified in other studies focusing on the general population include frustration and boredom—“exacerbated by not being able to take part in usual day-to-day activities, such as shopping for basic necessities” (Brooks et al., 2020, p. 115), fear of contamination and a lack of information, which were particularly present within our sample population. These pressures felt by the population could be intensified by a perceived feeling of increased isolation, as indicated by Rogers et al. (2021, p. 5) in their study about children with intellectual disabilities: “Mothers reported difficulty in providing the level of support for their children that they received prior to lockdown typically offered by external resources such as school, social services, respite, additional carers, family and friends. Most of these fell away when lockdown measures were implemented which evoked feelings of despondency.”

Over a third of those parents state that they were *not happy* during lockdown. Furthermore, in a study conducted within a paediatric unit in Lombardy, Fazzi and Galli (2020, p. 879) state that “children with neurodisabilities face additional challenges as the result of their functional limitation and changes to their daily routine. This situation adds further stress to parents already worried about infection. As Zahaika et al. (2021, p. 5) indicate, “caregiving is taking their strength” and most of them (88.5%) felt physically exhausted. Traumatic life events, such as a pandemic, can intensify experiences of stigma and discrimination and, due to national restrictions and denied access to services, families may feel abandoned by professional services (Rogers et al., 2021). Camden (2020), in his literature study and qualitative interviews conducted with eighteen parents, of which nine had children with neurodevelopmental disorders, highlights the importance of supporting the wellbeing of people who play an important role in these children’s lives, whose mental health is more at risk when they have a pre-existing behaviour or anxiety issue. This study highlights varying impacts among families, with “a few positive impacts […] stated by some parents, among which a reduction in social demands, more flexible hours, a chance to spend more time with their family and a reassessment of family priorities” (*op. cit.*, p. 4).

Parents within the sample expressed as many *negative feelings* as those of control families, but fewer of them described themselves as happy. We can set the assumption that, faced with lockdown, fatigue, irritation, stress, worry and anxiety have been felt equally by parents, regardless of their family situation, especially with young children under 6 years old, while perhaps also conveying the persistence of daily worries without the chance to have a break (Rogers et al., 2021) for families of children with special needs, rather than a better ability to adapt to this unique context among other families. Parents of children with neurodevelopmental disorders indicated that, during lockdown, they required more information regarding their children’s learning (50.54% of participants) than for their education (26.88%) and care (16.13%). Ehrler et al. (2021) also uncovered this anxiety regarding their child’s academic success [54 children born very preterm and 73 children with congenital heart disease; mean age: 10.4 (SD: 1.2) years] yet, in their study, this anxiety is significantly higher than among parents of their typically developing peers. Future qualitative studies could explore these feelings more in depth, for example by focusing on the type of disorder of the children.

### A Sudden Interruption in Medical Care

Medical care for the child could only be continued for less than a quarter of the families within our sample, matching the 22% estimated by Cacioppo et al. (2020) in their study of 1000 French parents with children who have a physical disability aged between zero and 18 (ECHO study). The authors add that: “The most frequent parental concern was the lack of rehabilitation during the lockdown. The purpose of regular rehabilitation is to maintain or progress motor skills and to prevent complications that could further alter mobility and increase difficulties in daily life, such as orthopaedic deformities or physical deconditioning. Therefore, the interruption or modification of medical care and rehabilitation could inevitably deteriorate the child’s physical status and functional ability” (*op. cit.*, p. 4). This interruption in care could lead to higher levels of stress for the parents due to their increased responsibilities (Zahaika et al., 2021).

Regarding access to healthcare, online consultation as a tool to carry on providing care was greatly appreciated (Bobo et al., 2020), yet scarcely mentioned in our study, probably due to the delays in authorising online rehabilitation care, despite the responsiveness of government decisions and of the Caisse Nationale d’Assurance Maladie (CNAM, French National Health Insurance Fund). Its impact was therefore spread over time, and might have benefited patients and families more after the first lockdown. For Camden (2020, p. 52), “setting up support and health care services, formally or informally, should be a priority; prioritising a technological shift by developing services including health care and follow-up care both in person and online (e.g., telehealth and tele-education) based on a collaborative approach on multiple levels could be a social innovation, carrying a great capacity to adapt to users’ and service providers’ various political, organisational, geographical, social and economic contexts.” It is, however, important to delve deeper into this issue by focusing on children with developmental disorders, as some studies did not find remote care to be as efficient for them as they were for adults (Chevance et al., 2020), and one can imagine the stress caused by a sudden stop in the care provided to their children and the additional isolation that can then be felt.

### Lockdown Rules Followed Significantly More Than in the Control Population

While in our study parents of children with developmental disorders have used social distancing measures considerably more than parents in the control population, we have also shown that single-parent families in our study have paid greater attention to protective measures (aside from social distancing rules; Moscaritolo et al., 2021). In both cases, these elements attest to additional worry and fear regarding the risk of contracting the virus in households where the parents’ presence is more than necessary when rehabilitation and care institutions are closed. Similar results were obtained in the study by Odeh et al. (2020), which focused on 235 Jordanian children with diabetes and showed that parents strictly followed social distancing rules in order to lower their anxiety. It seems to be the same for children, and the study by Ehrler et al. (2021) indicates that the parents of children with congenital heart disease were reported to be more concerned about becoming infected with SARS-CoV-2 than others. It would be interesting to specify in a later study whether there is a significant differentiation between the types of disorders (i.e., intellectual, physical disorders) or diseases in children whose parents feel more affected and have adopted these social distancing measures more than others, as was done by Burnett et al. (2021) regarding the level of distress among parents in Australia and Italy.

### Yet Strengthened Family Relationships

Surprisingly, and as opposed to what our hypothesis suggested, the results from this study evidence, for a majority of the families, more strengthening of family relationships than in the study population, even though tensions were present in a quarter of them, similarly to the control population. Not having to follow the usual rhythm of consultations and therapeutic follow-up, being among themselves and being able to look after their children most likely contributed to this improvement. The fact that the relationship between partners improved much more among the target population than among the general population is therefore particularly interesting, as it could show that parents are supporting each other more and acting as a team when faced with additional difficulties. This result should perhaps also be linked to their parenting role, perceived as more satisfying than within the population without special needs. The English mothers interviewed by Rogers et al. (2021) also reported “positive experiences such as reduced daily pressure and more opportunities for spending time as a family and enjoying hobbies and leisure activities” which can explain this strengthening in family relations. Thierry et al. (2020), in their study of the French general population of families with 8- to 9-year-old children (*n* = 4,877), state that the family’s protective environment lessened the impact of the pandemic, while this study only evidenced 16% of increased tensions for these families.

However, this improvement in family relationships should not distract from the perceived pressures from the environment (partner, family, friends, colleagues, educators or teachers, the media, social media, health professionals or social services) which added to the usual perceived mental load. This is consistent with a study by Cacioppo et al. (2020), which showed that 50% of parents stated their mental strain as the main struggle, and 60% a lack of support.

Further studies should explore in depth the coping strategies used by parents in stressful situations such as lockdown and look at whether some are more efficient than others in order to have a more relaxed experience of lockdown.

## Contributions of the Study

Our study looks at the subjective views of parents of young children with neurodevelopmental difficulties regarding their personal experience of lockdown, rather than the children’s experience and their additional disorders. Unlike Nonweiler et al. (2020) whose “Strengths and Difficulties Questionnaire” was addressed to the parents regarding their children’s experience, our study was not based on a standardised questionnaire. This was done in order to allow parents to answer as freely as possible. Our study provides some interesting elements, not only regarding their different socio-demographic profile, but also regarding the fact that they suffered more and felt more pressures than any other parents, yet they felt the same amount of negative feelings as them. The issue of the sudden interruption in care and a stronger adherence to protective measures against COVID-19 were also shown to be particularly important for those families. Finally, despite the additional tensions felt by those parents, the first lockdown seemed to have a beneficial impact on their family relations, especially on the relationship between partners, and like every other family they took pleasure in this family reunification. The results highlight the importance of listening to what these families have to say, rather than what we would like to hear from them.

## Limitations

Yet it is important to point out the use of a non-standardised questionnaire as a limitation to this study. We wished to gain a deep understanding of the way parents of young children experienced lockdown without increasing the time required to answer the questionnaire.

The representativeness of the participants could not be ensured in this study. However, the fact that a large number of responses from parents of children with (neuro)developmental disorders were received in just a few days shows how much these parents wished for their voices to be heard, and should therefore be taken in consideration. A qualitative study is planned in order to target a larger number of precarious parents who might not have access to the internet or might be less likely to answer open questions. The data was gathered during later lockdowns. The results of this study are currently being analysed, and we will test how much impact social determinants have on those results.

We didn’t ask for the children’s clinical diagnoses. Only the diagnoses reported by parents are available so that all we know is that one child in the family has one or more of the following (neuro)developmental disorders: motricity, language, learning, intellectual.

Finally, regarding our logistic regression models, our evaluation criterion consists in the presence of a child with special needs in a household compared to households without children with special needs, based on explanatory variables occurring during lockdown and therefore recent for them. A logistic regression would normally look at whether explanatory variables influence our evaluation criterion, for example whether the presence of pressures as felt by the respondent was significantly correlated with the presence of children with special needs.

## Conclusion

The 93 French families with at least one child under 6 years old and one with (neuro)developmental difficulties or a chronic illness suffered particularly from this lockdown, even though, for most of them, the chance to be with their family combined with a slower pace of life was felt as positive. The high number of such families who completed our online questionnaire shows how much they needed their voices to be heard and wished to maintain a social connection with the “outside” world.

Our study provides some interesting insights into the way they experienced this first lockdown in the COVID-19 pandemic. While it is not surprising that they would experience more pressure and suffering than general families, it can be noted that negative feelings such as fatigue, stress and worry are the same as in the control population. The interruption in the provision of care contributed both to a break in a fast-paced lifestyle, and an increase in these parents’ anxiety and isolation. Furthermore, while it did not affect the perceived satisfaction of their parenting role, it still appears urgent to prioritize the support and care for these families, who still required information, during those times of lockdown, by continuing to provide care to their children in one way or another and by ensuring that their need to adapt again does not exceed their own abilities and resources, especially as young children, with their high level of requirements, are within their home. The interruption in the care provided had suddenly changed the rhythm of a usually fast-paced lifestyle and increased anxiety and isolation among those parents despite the fact that they still perceived their parenting role as satisfying. Given the cyclic nature of this still active pandemic, supporting and accompanying families of young children with a disability or developmental disorders must be a priority.

Public policies should ensure, one way or another, that the provision of care for these children is not interrupted, not only to ensure their continued development in the best possible conditions but also to ensure that the parents’ abilities, resources and mental load, which are already tested in normal circumstances, do not wear out any further. In light of the results, several supportive interventions for families and children with (neuro)developmental disorders could be suggested: offering “online” care appears to be more than necessary in order to provide advice and support to those families. As Rogers et al. (2021) suggested, neighbours could be encouraged through national and local media to support these families by looking after young children, for example by reading a daily story *via* a video call or by providing regular support over the phone, and to ensure that they feel socially included. This support could help parents be in a better mood, which could in turn lead to better outcomes for the children. In France just like in many other western countries, the pandemic has been an unpredictable event, prompting families to seek help with little support from health authorities at a time when the main focus was devoted to responding to emergency situations. Thus, our work was intended to provide insights from sociological research in order to support the public response and health policies for any future and unexpected events affecting children with special needs and their families.

The general presentation of our results could lead one to forget how things can vary from one family to another. We should point out that the parents did not provide a description of the disorders or their intensity. The disabilities or difficulties could therefore vary greatly between families. Yet, while families showed more negative feelings toward the effects of lockdown, some had a positive experience of being able to spend time as a family. Similarly, in their study conducted with children with an attention deficit hyperactivity disorder (ADHD), Bobo et al. (2020) state that, overall, they have experienced either greater wellbeing or a stable psychological condition with lowered anxiety due to the interruption of the school rhythm and a “tailored” lifestyle. Cacioppo et al. (2020, p. 6) reminds us that “one of the main results of the ECHO survey was that the effects and experiences of the lockdown differed among families. This finding demonstrates the importance of assessing each situation individually to maintain and promote quality of life in children and their families, including during and after the lockdown.”

This variability can also apply to differences in the care (continuity/discontinuity), and Toseeb et al. (2020) confirmed in their online study of 339 parents in the United Kingdom, among which 81% have autistic children, that parents’ satisfaction regarding the support provided to their children varied greatly, which could, according to them, suggest inequalities in the support provided.

## Data Availability Statement

The original contributions presented in the study are included in the article/Supplementary Material, further inquiries can be directed to the corresponding author/s.

## Ethics Statement

This study was approved by the General Data Protection Regulation’s Data Protection Officer [RGPD-CNRS (TRRECH-502)] and by the research ethics board (CER; number 2020-290) from the Université Fédérale de Toulouse Midi-Pyrénées. The patients/participants provided their written informed consent to participate in this study.

## Author Contributions

All authors listed have made a substantial, direct, and intellectual contribution to the work, and approved it for publication.

## Conflict of Interest

The authors declare that the research was conducted in the absence of any commercial or financial relationships that could be construed as a potential conflict of interest.

## Publisher’s Note

All claims expressed in this article are solely those of the authors and do not necessarily represent those of their affiliated organizations, or those of the publisher, the editors and the reviewers. Any product that may be evaluated in this article, or claim that may be made by its manufacturer, is not guaranteed or endorsed by the publisher.

## Footnotes

1 For the COV-JEUNENFANT Consortium Chantal Zaouche Gaudron (Ed.), Jean-Charles Basson, Claire Bouilhac, Isabelle Claudet, Natacha Collomb, Cyrille Delpierre, Anne Dupuy, Emilie Gaborit, Isabelle Glorieux, Valérie Katkoff, Michelle Kelly-Irving, Valérie Larrosa, Muriel Lefebvre, Pascal Marchand, Christine Mennesson, Alice Moscaritolo, Stéphanie Pinel-Jacquemin, Pierre Ratinaud, Julie Renard, Thiébaut-Noël Willig. Financed by: Agence Nationale Régionale, Région Occitanie, Caisse Nationale Allocations Familiales, Caisse Allocations Familiales 31, Conseil Départemental 31 ANR-20-COV1-000.

2 Question 10 specified: developmental disorder (motricity, language, learning, intellectual)

3 Apart from the 40 families who reported having a child/children with a DD, 53 families have answered “yes” to item 8: “Is one of your children usually receiving care at or outside the home for health issues (general medicine, speech therapy, physiotherapy, psychomotricity…) apart from routine medical visits?” *Two families have two children with a DD (children 1 and 2 for one of them, and children 2 and 3 for the other).

4
https://pinel-jacquemin.wixsite.com/covjenfant

5 CNRS: Centre National de Recherche Scientifique: https://sms.univ-tlse2.fr/parcours-de-vie-et-reseaux-personnels-/enquete-relations-sociales-et-solidarites-en-periode-de-confinement-confinement–729547.kjsp?RH=ACCUEIL_SMS).
