# Supplementary material for: Lockdown in France: Impact on Families of Young Children With Special Needs
Source: Front Psychol. 2022 Apr 26;13:781030. doi: 10.3389/fpsyg.2022.781030 (PMC9088877; doi:10.3389/fpsyg.2022.781030)
Supplement: Supplementary file 1 [file Data_Sheet_1.pdf]

Appendix 1: Verifying statistically relevant results from the chi-square test from table 2

As Sharpe and Donald (2015) point out, when the result of a chi-square test is associated with a higher number of degrees of freedom, the source of a statistically relevant result should be checked, for example through the residuals from the difference between observed and expected data.

<u>Number of children in the household</u>:

**Number of children in the household**	**Population without 'Special needs'**	**'Special needs' population**
**1 child**		
Observed rate	157	26
Expected rate	148.267	34.733
Raw residuals	8.733	-8.733
Pearson residuals	0.717	-1.482
Chi-square contributions	0.514	2.196
**2 children**		
Observed rate	171	36
Expected rate	167.712	39.288
Raw residuals	3.288	-3.288
Pearson residuals	0.254	-0.525
Chi-square contributions	0.064	0.275
**3 children and more**		
Observed rate	69	31
Expected rate	81.020	18.980
Raw residuals	-12.020	12.020
Pearson residuals	-1.335	2.759
Chi-square contributions	1.783	7.613

Pearson chi2(2) = 12.4458 Pr = 0.002 likelihood-ratio chi2(2) = 11.5152 Pr = 0.003

<u>Partner's education level</u>:

Partner's education level	Population without 'Special needs'	'Special needs' population
High school diploma or lower		
Observed rate	60	25
Expected rate	68.867	16.133
Raw residuals	-8.867	8.867
Pearson residuals	-1.069	2.208
Chi-square contributions	1.142	4.874
Less than 4 years after high school diploma		
Observed rate	106	25
Expected rate	106.137	24.863
Raw residuals	-0.137	0.137
Pearson residuals	-0.013	0.027

Chi-square contributions	0.000	0.001
4 years or more after high school diploma		
Observed rate	209	32
Expected rate	195.259	45.741
Raw residuals	13.741	-13.741
Pearson residuals	0.983	-2.032
Chi-square contributions	0.967	4.128
Missing		
Observed rate	22	11
Expected rate	26.737	6.263
Raw residuals	-4.737	4.737
Pearson residuals	-0.916	1.893
Chi-square contributions	0.839	3.582

Pearson chi2(3) = 15.5329 Pr = 0.001 likelihood-ratio chi2(3) = 14.7347 Pr = 0.002

Income based on consumption units (INSEE)

**Income terciles based on consumption units (INSEE)**	**Population without 'Special needs'**	**'Special needs' population**
**Median income 1st tercile (1257.56€)**		
Observed rate	122	43
Expected rate	133.684	31.316
Raw residuals	-11.684	11.684
Pearson residuals	-1.011	2.088
Chi-square contributions	1.021	4.359
**Median income 2nd tercile (1976.19€)**		
Observed rate	166	38
Expected rate	165.282	38.718
Raw residuals	0.718	-0.718
Pearson residuals	0.056	-0.115
Chi-square contributions	0.003	0.013
**Median income 3rd tercile (2857.14€)**		
Observed rate	109	12
Expected rate	98.035	22.965
Raw residuals	10.965	-10.965
Pearson residuals	1.107	-2.288
Chi-square contributions	1.226	5.236

Pearson chi2(2) = 11.8587 Pr = 0.003 likelihood-ratio chi2(2) = 12.5045 Pr = 0.002

Financially comfortable:

**Financially comfortable**	**Population without 'Special needs'**	**'Special needs' population**
**Not at all**		
Observed rate	67	28
Expected rate	76.969	18.031
Raw residuals	-9.969	9.969
Pearson residuals	-1.136	2.348
Chi-square contributions	1.291	5.512
**Relatively**		
Observed rate	236	48
Expected rate	230.098	53.902
Raw residuals	5.902	-5.902
Pearson residuals	0.389	-0.804
Chi-square contributions	0.151	0.646
**Very**		
Observed rate	94	17
Expected rate	89.933	21.067
Raw residuals	4.067	-4.067
Pearson residuals	0.429	-0.886
Chi-square contributions	0.184	0.785

Pearson chi2(2) = 8.5703 Pr = 0.014 likelihood-ratio chi2(2) = 7.9035 Pr = 0.019

For all of these results, the cells produced residuals of +/-2 (no cell produced a residual above +/-3).

Appendix 2: Verifying statistically relevant results from the chi-square test from table 3

Score for pressures felt during lockdown:

Score for pressures felt	Population without ‘Special needs’	‘Special needs’ population
**no pressures**		
Observed rate	158	23
Expected rate	146.647	34.353
Raw residuals	11.353	-11.353
Pearson residuals	0.938	-1.937
Chi-square contributions	0.879	3.752
**1 or 2 slight pressures or 1 strong pressure**		
Observed rate	137	36
Expected rate	140.165	32.835
Raw residuals	-3.165	3.165
Pearson residuals	-0.267	0.552
Chi-square contributions	0.071	0.305
**more than 1 strong pressure or more than 2 slight pressures**		
Observed rate	102	34
Expected rate	110.188	25.812
Raw residuals	-8.188	8.188
Pearson residuals	-0.780	1.612
Chi-square contributions	0.608	2.597

Pearson chi2(2) = 8.2131 Pr = 0.016 likelihood-ratio chi2(2) = 8.4593 Pr = 0.015

Respondent’s free time during lockdown:

Free time	Population without ‘Special needs’	‘Special needs’ population
**no, I don’t lack free time at all**		
Observed rate	92	12
Expected rate	84.261	19.739
Raw residuals	7.739	-7.739
Pearson residuals	0.843	-1.742
Chi-square contributions	0.711	3.034
**yes, I really lack free time**		
Observed rate	170	53
Expected rate	180.676	42.324
Raw residuals	-10.676	10.676
Pearson residuals	-0.794	1.641
Chi-square contributions	0.631	2.693

yes, I somewhat lack free time		
Observed rate	135	28
Expected rate	132.063	30.937
Raw residuals	2.937	-2.937
Pearson residuals	0.256	-0.528
Chi-square contributions	0.065	0.279

Pearson chi2(2) = 7.4124 Pr = 0.025 likelihood-ratio chi2(2) = 7.7108 Pr = 0.021

Changes in relationships within the family during lockdown:

Relationships within the family	Population without 'Special needs'	'Special needs' population
**tense**		
Observed rate	95	24
Expected rate	96.414	22.586
Raw residuals	-1.414	1.414
Pearson residuals	-0.144	0.298
Chi-square contributions	0.021	0.089
**unchanged**		
Observed rate	143	20
Expected rate	132.063	30.937
Raw residuals	10.937	-10.937
Pearson residuals	0.952	-1.966
Chi-square contributions	0.906	3.866
**strengthened**		
Observed rate	159	49
Expected rate	168.522	39.478
Raw residuals	-9.522	9.522
Pearson residuals	-0.734	1.516
Chi-square contributions	0.538	2.297

Pearson chi2(2) = 7.7164 Pr = 0.021 likelihood-ratio chi2(2) = 8.0945 Pr = 0.017

Perception of parental role during lockdown:

Parental role	Population without 'Special needs'	'Special needs' population
**Satisfying**		
Observed rate	214	65
Expected rate	226.047	52.953
Raw residuals	-12.047	12.047
Pearson residuals	-0.801	1.656
Chi-square contributions	0.642	2.741

**Neutral**		
Observed rate	108	18
Expected rate	102.086	23.914
Raw residuals	5.914	-5.914
Pearson residuals	0.585	-1.209
Chi-square contributions	0.343	1.463
**Unsatisfying**		
Observed rate	75	10
Expected rate	68.867	16.133
Raw residuals	6.133	-6.133
Pearson residuals	0.739	-1.527
Chi-square contributions	0.546	2.331

Pearson chi2(2) = 8.0654 Pr = 0.018 likelihood-ratio chi2(2) = 8.3752 Pr = 0.015

For all of these results, the cells produced residuals of +/-2 (no cell produced a residual above +/-3).
