# Supplementary material for: Lockdown in France: Impact on Families of Young Children With Special Needs
Source: Front Psychol. 2022 Apr 26;13:781030. doi: 10.3389/fpsyg.2022.781030 (PMC9088877; doi:10.3389/fpsyg.2022.781030)
Supplement: Supplementary file 2 [file Data_Sheet_2.docx]

**Additional material : « COVJEUNENFANT » Questionnaire**

The questionnaire was made of 69 questions as follows:

1. **General information (questions 1 to 18) :**

Socio-demographic data, children’s specific medical or paramedical care, continuity of current care and the existence of a developmental disorder (motricity, language, school learning, intellectual) in one of the children.

1. **Parents’ experience of the pandemic (questions 19 to 26)**

An open question invites the participant to express themselves freely with regards to this “unusual” time in order to better understand how they and their young children experienced this lockdown (in terms of activities, play, screen time, diet, schoolwork, education in a broad sense…) and how they felt (Q19). The following questions asked them to describe “four main events” in “yesterday’s day in lockdown” (Q20), specifying for each event whether they were alone or with someone, and if so who, during that day (Q21), and specifying how they felt among a list of options (Looking forward to finishing; Happy; Frustrated/annoyed; Depressed; Feeling competent/qualified; Feeling mistreated/abused; Warmth/friendship; Anger/hostility; Worried/anxious; Feeling pleased; Feeling criticised; Fatigue) and how intensely they felt the emotion (from not at all to a lot) (Q22-25). Question 26 allowed us to learn how often (from never to often) they found it hard to control important things in their lives, if they felt confident in their ability to handle their personal problems, if they felt like things were going as they wanted and if they felt as though difficulties were accumulating to the point where they could not handle them.

1. **Child’s self-expression (question 27)**

“If one (or more) of your children under 6 years old wishes to, they can answer as freely as possible, through a word, a sentence or more, to the following question: “Compared to your life right now, what would you say about it?” It is important that your child/children is/are allowed to express themselves freely, and only if they can and wish to do so. Please specify the age of the child answering the question.

Please write what your child (under 6 years old) says, trying not to change their words, even if it is only individual words or short fragments of a sentence.

Please feel free to use as much space as needed.

The phrasing or potential mistakes do not matter.

1. **Activities and daily life during lockdown (questions 28 to 46)**

Question 28 looks at the presence of Covid symptoms among the participants or the people close to them during this period (cough, headaches, fever, loss of smell or taste, difficulty breathing, others). Whether those symptoms were benign, severe but did not require treatment at a hospital, severe and required treatment at the hospital, or severe and required treatment at the hospital in intensive care (Q29). Whether they had other health issues apart from COVID (Q30, open question), whether they were able to get some support in case the person suffering from COVID was the participant (Housework; Preparing meals; Children’s hobbies; Checking children’s homework; Caring for the children; Groceries shopping; Other activities: specify in a comment) (Q31).

Question 32 looked at activities outside working hours (children’s education, housework, etc.). Question 33 asked to specify whether these tasks differed from those before lockdown.

The change in how children’s education (help with homework, children’s hobbies, children’s care, etc.) and housework (groceries, meals, housekeeping and laundry, etc.) were shared between the participants and their partners during lockdown is covered by Q34.

Question 35 covers the measures taken by the participants to protect themselves from being contaminated with COVID (Do you wear gloves? Do you wear a mask? Do you wear coveralls? Do you keep at least a one meter distance when meeting someone outside? Do you wash your hands several times a day? Do you disinfect your hands with antibacterial gel several times a day? Do you wash the clothes worn outside home? Do you disinfect door handles, switches and other potentially contaminated surfaces? Other, please specify in a comment).

Measures taken to protect children from contamination (Q36, same list).

Q37. Average time (not including weekends and school holidays) dedicated to helping your children with their schoolwork or home-teaching and cultural activities (crafts, sports, reading, games…) to be specified among a list of available options (1 = I don’t have any children in school, 2 = Up to 2 hours per day, 3 = Between 2 and 4 hours per day, every day of the week or nearly, 4 = Between 4 and 6 hours per day, every day of the week or nearly, 5 = Over 6 hours per day, every day of the week or nearly).

Q38. Same question for the participant’s partner.

Q39. Feel like they lack free time (Yes, I really lack free time; Yes, I somewhat lack free time; No, I don’t really lack free time; No, I don’t lack free time at all).

Q40. Time(s) when the participant felt Tired, Irritated, Relaxed, Energetic, Stressed, Sad, Happy, Worried, Anxious, None, Other: please specify in a comment.

Q41. On average and excluding grocery-shopping, how often did the participant leave their home to get some fresh air, walk or exercise (Several times per day, everyday or almost every day; Once per day, every day or almost every day; Several times per week, but not every day; Around once per week; Less than once per week; Never).

Q42. Who did they go out with (Alone most of the time; With one or more people with whom you were isolating; With one or more people you met outside; It depends, sometimes alone and sometimes with one or more people).

Q43. Looks at how relations between members of the family changed during lockdown (No, they did not change; Yes, the relationship between me and my partner became more tense; Yes, the relationship between parents and children became more tense; Yes, the relationship between me and my partner became stronger; Yes, the relationship between parents and children became stronger).

Q44. Whether they felt pressured in the way they looked after their children (A little, A lot, Not at all): If in a relationship, from your partner; If in a relationship, from your family or your partner’s family; If shared custody, from your ex-partner or their family; From some members of the family (siblings, parents, uncles, aunts…); From your friends or colleagues; From educators or teachers; From the media (newspapers, TV…); From social media (online forums, specialised websites, Facebook…); From healthcare professionals or social workers.

Q45. Agree or disagree (from 1 = Strongly disagree, 2 = Disagree, 3 = Neither agree nor disagree, 4 = Agree, 5 = Strongly agree) with the following statements: I feel like I am not doing enough to stimulate my children; I feel like I am being asked to organise educational activities for my children for which I do not feel competent; I fear that my children might suffer in the long term from the lack of stimulation or educational activities; I feel overwhelmed by all the things I need to do to look after my children; I tell myself that I am the best person to tell what is good for my children.

Q46. Need for information since lockdown regarding education (respecting rules, setting limits, etc.), care for young children (diet, hygiene, health, etc.) and learning.

1. **Accommodation during lockdown (questions 47 to 58)**

Did the participant leave their usual accommodation (Q47)? Were they in their main or secondary residence (Q48), in which county (Q49), in which municipality (Q50), what type of accommodation (Q51) (House, Apartment, Other (hotel room or student accommodation, mobile home, etc.) please specify in a comment), the number of rooms (Q52), access to outdoors areas (Q53) (Balcony; Garden or exclusive use of a piece of land; Exclusive use of a yard or patio; Shared yard or garden (building, gated residence, etc.); No access to an outdoor space), the type of neighbouring accommodations (Q54) (Spread out houses in rural area; Housing estate in residential area or in a city; Building in a city (other than building complex); Building in a building complex; Mixed types of housing: both buildings and houses). The number of people living in the accommodation at the time of answering (Q55) and who those people are (Q56) (My partner; One or more of my children or my partner’s children; One or more of my grandchildren or my partner’s grandchildren; One or more of my parents (father, mother) or my partner's parents; One or more of my grandparents or my partner's grandparents; One or more other members of my family (brother, sister, uncle, aunt, cousin, brother or sister in-law, son or daughter in-law…) or my partner’s family; One or more friends; Other people, please specify in a comment). Whether there is a room where the participant can be alone, in a quiet area (Q57), and the problems faced within this environment (Q58) (Pollution outside the accommodation; Lack of security outside the accommodation; Dirty streets or neighbourhood; Difficulty in finding some essential staple foods; Noise pollution outside the accommodation (noise from the street, the neighbours); Tensions or conflict with neighbours; Noise pollution inside the accommodation (noise from other people living there); Tensions or conflict with other people living in the accommodation; Lack of space within the accommodation; None of these problems).

1. **Employment since the beginning of lockdown (questions 59 to 68)**

The profession or employment status of the participant (Q59), of their partner (Q60), occupational category of the participant and their partner (Q61), the number of hours worked by the participant (Q62) and their partner (Q63), their partner’s main employment status (Q64) (Employed by a company, a craftsman, a merchant; Employed, civil servant or public company; Employed by a charity, a foundation or a non-governmental organisation; In work, as self-employed; Apprenticeship contract or paid internship; Studying (student, pupil, in training or unpaid internship); Retired or early-retirement; Homemaker). Whether the participant was (Q65): Made redundant; Put on leave for one or more days; Temporary unemployment; Reduction in working hours (for employees) or in business (for self-employed); Return to work after being off work, on holiday or unemployed before the start of lockdown; Increase in mandatory working hours; Obligation to work unusual hours; Deterioration of working conditions; Significant decrease of income; None of the above; and same question for their partner (Q66). The place of work (Q67) (Solely at a place of work or study outside the home where they were isolating; Mainly in a place of work or study outside the home, but also partly within the accommodation where they were isolating; Mainly within the accommodation where they were isolating, but also partly in a place of work or study outside the home; Solely in the home where they were isolating; Didn’t work) and same question for their partner (Q68).

**Question 69** was an open question inviting respondents to add any comments which they felt were important.
